# Serum markers of microbial translocation and intestinal damage in assessment of gastrointestinal tract involvement in systemic sclerosis

**DOI:** 10.1007/s10238-024-01466-1

**Published:** 2024-09-19

**Authors:** Chiara Pellicano, Alessandra Oliva, Amalia Colalillo, Antonietta Gigante, Elisa D’Aliesio, Dania Al Ismail, Maria Claudia Miele, Rosario Cianci, Claudio Maria Mastroianni, Edoardo Rosato

**Affiliations:** 1 Department of Translational and Precision Medicine, Sapienza University of Rome, Viale dell’Università 37, 00185 Rome, Italy; 2 Department of Public Health and Infectious Diseases, Sapienza University of Rome, 00185 Rome, Italy

**Keywords:** Systemic sclerosis, Gastrointestinal, UCLA, LBP, Zonulin, Microbial translocation

## Abstract

Gastrointestinal (GI) tract involvement affects up to 90% of Systemic sclerosis (SSc) patients. The presence of GI symptoms is assessed by the University of California, Los Angeles, and Scleroderma Clinical Trials Consortium Gastrointestinal Scale (UCLA SCTC GIT 2.0). Microbial translocation (MT) is reported in SSc patients consequently to increased intestinal permeability due to intestinal damage (ID) and dysbiosis. Aim of this study was to assess circulating levels of LBP and EndoCab IgM (markers of MT), IL-6 (marker of inflammation), I-FABP and Zonulin (markers of ID) in a cohort of SSc patients and healthy controls (HC). Moreover, we aimed to correlate these parameters with severity of GI symptoms. UCLA SCTC GIT 2.0 questionnaire was administered to 60 consecutive SSc patients. Markers of MT, inflammation and ID were evaluated in SSc patients and HC. SSc patients had higher median value of markers of MT, inflammation and ID than HC. The logistic regression analysis showed LBP as the only variable associated with an UCLA total score “moderate-to-very severe” [OR 1.001 (CI 95%: 1.001–1.002), *p* < 0.001]. The logistic regression analysis showed LBP [OR 1.002 (CI 95%: 1.001–1.003), *p* < 0.01] and disease duration [OR 1.242 (CI 95%: 1.023–1.506), *p* < 0.05] as variables associated with UCLA distension/bloating “moderate-to-very severe”. The logistic regression analysis showed LBP as the only variable associated with UCLA diarrhea “moderate-to-very severe” [OR 1.002 (CI 95%: 1.001–1.003), *p* < 0.01]. SSc patients with dysregulation gut mucosal integrity expressed by high levels of MT and ID biomarkers had more severe GI symptoms.

## Introduction

Systemic sclerosis (SSc) is a complex systemic autoimmune disease characterized by vasculopathy, immune activation and fibrosis of skin and internal organ [1]. Gastrointestinal (GI) tract involvement affects up to 90% of SSc patients and is manifested primarily as dysmotility, due to a progression of vasculopathy, myopathy, neuropathy and fibrosis leading to abnormalities in compliance and contractility of the GI tract wall [2, 3]. Any segment of the GI tract may be affected and 98.9% of SSc patients reported GI symptoms, including dysphagia, heartburn, and distention/bloating, abdominal pain, nausea, vomiting, diarrhea, constipation and fecal incontinence [4]. Local complications can develop as a result of dysmotility including reflux esophagitis and small intestinal bacterial overgrowth (SIBO), expression of gut dysbiosis [5]. The presence of GI symptoms and their impact on quality of life (QoL) of SSc patients are assessed by the University of California, Los Angeles, and Scleroderma Clinical Trials Consortium Gastrointestinal Scale (UCLA SCTC GIT 2.0) [6].

Dysbiosis is associated with an alteration in the intestinal barrier integrity, which leads to a subsequent passage of microorganisms, or their products, into the systemic circulation, a phenomenon known as microbial translocation (MT) and described during infective and non-infective conditions [7–20]. Experimental and clinical studies demonstrated that Zonulin upregulation plays a key role in increasing gut permeability, by disassembling the intercellular tight junctions, and that serum levels of Zonulin correlate with enhanced intestinal permeability [21]. Moreover, intestinal fatty acid binding protein (I-FABP) is recognized as a marker of ID, since it is released as soon as cell membrane integrity of enterocytes in the small intestine is compromised and subsequently appears in the circulation only after enterocyte injury [22]. Increased gut permeability is responsible of MT and even a low-grade endotoxemia may induce production of host response molecules such as lipopolysaccharide-binding protein (LBP) and consumption of neutralizing antibodies against lipopolysaccharide (LPS) endotoxin core antigen [antiendotoxin core antibody (EndoCab)] [23]. Moreover, it is well known that circulating LPS is a potent proinflammatory factor that may exacerbate inflammatory responses with production of several inflammatory cytokines such as interleukin (IL)-6 [24].

To date, only one study investigated intestinal permeability in SSc patients, suggesting that increased intestinal permeability in SSc may exacerbate the course of the disease and increase the risk of developing complications [25].

Based on these premises, and trying to filling the knowledge gap in this filed, the aim of this study was to assess circulating levels of LBP and EndoCab IgM (markers of MT), IL-6 (marker of inflammation), I-FABP and Zonulin (markers of ID) in a cohort of SSc patients and healthy controls (HC). Moreover, we aimed to correlate these parameters with severity of GI symptoms reported through the UCLA SCTC GIT 2.0 questionnaire in SSc patients.

## Methods

### Subjects

In this monocentric cross-sectional study, we enrolled 60 consecutive SSc patients, fulfilling the 2013 American College of Rheumatology/European League Against Rheumatism Collaborative Criteria (ACR/EULAR) for SSc [26], and 20 HC matched for sex and age and recruited among healthcare workers.

Exclusion criteria for both SSc patients and HC were: acute or chronic GI infection in the last three months, history of inflammatory bowel disease (IBD), concomitant or previous malignancy, GI surgery or loss of weight in the last six months, treatment with antibiotics or probiotics or prebiotics in the last three months. Smokers, pregnant or breastfeeding women and patients treated in the last six months with nonsteroidal anti-inflammatory drugs (NSAID), immunosuppressive agents and corticosteroids at an equivalent dose of prednisone ≥ 10 mg/day were also excluded. Each subject enrolled was asked whether he/she suffered from diarrhea in the 30 days before the blood collection or other chronic intestinal issues or new-onset symptoms. In the presence of at least one of these conditions, subjects were excluded from the study.

The subjects' written consent was obtained and the study was conducted according to the Declaration of Helsinki. The study was approved by the ethics committee of Sapienza University (IRB n 394/17).

### Clinical assessment

According to Le Roy et al. [27], disease subset was defined as limited cutaneous (lc)SSc or diffuse cutaneous (dc)SSc and the modified Rodnan skin score (mRSS) was assessed by the same experienced operator blinded of laboratory assessment and of other clinical characteristics of SSc patients. Disease duration (time from first non-Raynaud manifestation), disease activity index (DAI) and disease severity scale (DSS) were assessed following the European Scleroderma Trials and Research (EUSTAR) group indications [28, 29]. Nailfold videocapillaroscopy (NVC) was performed in both hands at the level of the distal phalanx of the second, third and fourth fingers with a videocapillaroscope equipped with a 200x magnification lens (VideoCap 3.0, DS Medica, Italy) and the capillaroscopic patterns were classified in early, active, and late, according to Cutolo et al. [30]. NVC was performed after resting the subject in a temperature controlled room at 24 ± 0.4 °C for 20 min by the same experienced operator blinded of laboratory assessment and of other clinical characteristics of SSc patients.

### University of California, Los Angeles Scleroderma Clinical Trial Consortium GIT 2.0 (*UCLA* SCTC GIT 2.0, *UCLA*)

UCLA SCTC GIT 2.0 questionnaire was administered to all patients by an experienced specialist blinded of laboratory assessment and of other clinical characteristics of SSc patients. This questionnaire contains seven specific scales (reflux, distention/bloating, diarrhea, fecal soilage, constipation, emotional well-being, and social functioning) with 34 items on symptoms of GI tract involvement and the impact on QoL [6]. The scales are scored from 0.0 (better QoL) to 3.0 (worse QoL). Only diarrhea and constipation scales range from 0.0 to 2.0 and 0.0 to 2.5, respectively. The total score is obtained from average of six of seven scales (except constipation) and it is scored from 0.0 (better QoL) to 3.0 (worse QoL). The severity for each reported score was define as “none-to-mild”, “moderate” and “severe-to-very severe”, according to Khanna et al. [31].

### Laboratory assessment

Markers of MT (LPB, Hycult Biotech, https://www.hycultbiotech.com/product/lbp-human-elisa-kit/; EndoCab IgM, Hycult Biotech, https://www.hycultbiotech.com/product/hk504-igm/), inflammation (IL-6, R&D Systems, https://www.rndsystems.com/target/il6?category%20=%20ELISAs&gad_source%20=%201&gclid%20=%20CjwKCAjwnqK1BhBvEiwAi7o0XxbBVnPuODDtiEXtS6tprT6Dgy5Q1-FwDK_WHeWPWn570c2_UrsLChoC_n8QAvD_BwE&gclsrc%20=%20aw.ds), ID (I-FABP, R&D Systems, https://www.rndsystems.com/target/fabp2-i-fabp; Zonulin, Elabscience, https://www.elabscience.com/p-human_zonulin_elisa_kit41796.html?&utm_campaignid%20=%2010832611886&utm_adgroupid%20=%20108509122736&utm_creative%20=%20662278143503&utm_network%20=%20g&utm_matchtype%20=%20&utm_device%20=%20c&utm_devicemodel%20=%20&utm_term%20=%20&utm_adposition%20=%20&utm_placement%20=%20&utm_feeditemid%20=%20&utm_targetid%20=%20dsa437115340933&gad_source%20=%201&gclid%20=%20CjwKCAjwnqK1BhBvEiwAi7o0XyE68kPo0blISdIj5py9d8apFsHUrGYkw5zwLFhBp7cgJv185t1LGxoCRxUQAvD_BwE) were evaluated throughout ELISA assays in plasma, according to the manufacturer's instructions and previous studies from our group [19–21, 32].

Briefly, after collection blood was immediately centrifuged at 2000 rpm for 10 min and further frozen at −80 °C until the assays were performed. LBP was expressed as ng/ml (sample diluted 1:1000), EndoCab IgM as MU/ml (sample diluted 1:50), IL-6 as pg/ml (sample diluted 1:2), Zonulin (sample undiluted) and I-FABP as pg/ml (sample diluted 1:5). Laboratory operators were all blind to the clinical information throughout the study.

### Statistical analysis

SPSS version 25.0 software (Bioz, Los Altos, CA) was used for statistical analysis. After evaluation of normality, continuous variables were expressed as median and interquartile range (IQR) and categorical variables were expressed as absolute frequency and percentage (%). Student's *t*-test or Mann–Whitney's *U*-test was used to evaluate differences between groups, as appropriate. Bonferroni’s corrections were applied in case of multiple comparisons. The chi-square or Fisher’s exact test was used to evaluate differences between categorical variables, as appropriate. The 2-tails Pearson or Spearman’s correlation test was used for bivariate correlations. Stepwise logistic regression analysis was used to evaluate the association between a dependent dichotomous variable (UCLA total score “moderate-to-very severe” or UCLA distension/bloating “moderate-to-very severe” or UCLA diarrhea “moderate-to-very severe”) and continuous independent variables which were significant at the univariable analysis for each dependent variable. Results were expressed as odds ratio (OR) and 95% confidence interval (95% CI). *P* value < 0.05 was considered significant.

## Results

### Demographic and clinic characteristics of SSc patients

Forty-nine (81.7%) patients were females. Median age was 55 years (IQR 48–63 years). Thirty-three (55%) patients had dcSSc and 27 (45%) had lcSSc, with a median mRSS of 12 (IQR 7–20). Demographic and clinical features of SSc patients are shown in Table 1.

**Table 1:** Demographic and clinical features of systemic sclerosis (SSc) patients

Age, years, median and IQR	55 (48–63)
Female, *n* (%)	49 (81.7)
dcSSc, *n* (%)	33 (55)
Disease duration, years, median and IQR	13 (7–19)
mRSS, median and IQR	12 (7–20)
SSc-specific Autoantibodies	
Anti-topoisomerase I, *n* (%)	26 (43.3)
Anti-centromere, *n* (%)	13 (21.7)
Anti-RNApolimerase III, *n* (%)	4 (6.7)
None, *n* (%)	17 (28.3)
NVC	
Early, *n* (%)	9 (15)
Active, *n* (%)	16 (26.7)
Late, *n* (%)	35 (58.3)
DAI, median and IQR	2.3 (1.09–3.87)
DSS, median and IQR	6 (4-9)

SSc: Systemic Sclerosis; dcSSc: Diffuse Cutaneous Systemic Sclerosis; mRSS: Modified Rodnan Skin Score; NVC: Nailfold Videocapillaroscopy; DAI: Disease Activity Index; DSS: Disease Severity Scale; IQR: Interquartile Range

All patients reported at least one symptom of GI involvement. According to the UCLA SCTC GIT 2.0 questionnaire, 21 of 60 (35%) patients had a total score compatible with severe GI symptoms; 28 of 60 (46.7%) had severe reflux; 23 of 60 (38.3%) had severe abdominal distension/bloating symptoms; 26 of 60 (43.3%) had severe diarrhea; none (0%) had severe fecal soilage; 2 of 60 (3.3%) had severe constipation; 8 of 60 (13.3%) had severe problems in the emotional well-being and 17 of 60 (28.3%) had severe limitations in social function. Table 2 shows the UCLA SCTC GIT 2.0 total score and single items.

**Table 2:** Results of University of California, Los Angeles Scleroderma Clinical Trial Consortium GIT 2.0 (UCLA SCTC GIT 2.0, UCLA) questionnaire of systemic sclerosis (SSc) patients

Total score, median and IQR	0.708 (0.471–1.181)
Total score gravity, *n* (%)	
None-to-mild (0.00–0.49)	20 (33.3)
Moderate (0.50–1.00)	19 (31.7)
Severe-to-very severe (1.01–3.00)	21 (35)
Reflux, median and IQR	1 (1–1.5)
Reflux gravity, *n* (%)	
None-to-mild (0.00–0.49)	2 (3.3)
Moderate (0.50–1.00)	30 (50)
Severe-to-very severe (1.01–3.00)	28 (46.7)
Distension/bloating, median and IQR	1.5 (0.85–1.8)
Distension/bloating gravity, *n* (%)	
None-to-mild (0.00–1.00)	20 (33.3)
Moderate (1.01–1.60)	17 (28.3)
Severe-to-very severe (1.61–3.00)	23 (38.3)
Diarrhea, median and IQR	0.9 (0.1-1.4)
Diarrhea gravity, *n* (%)	
None-to-mild (0.00–0.49)	22 (36.7)
Moderate (0.50–1.00)	12 (20)
Severe-to-very severe (1.01–2.00)	26 (43.3)
Fecal soilage, median and IQR	0 (0-0.7)
Fecal soilage gravity, *n* (%)	
None-to-mild (0.00–1.00)	56 (93.3)
Moderate (1.01–2.00)	4 (6.7)
Severe-to-very severe (2.01–2.50)	0 (0)
Constipation, median and IQR	0 (0-0.5)
Constipation gravity, *n* (%)	
None-to-mild (0.00–0.49)	42 (70)
Moderate (0.50–1.00)	16 (26.7)
Severe-to-very severe (1.01–3.00)	2 (3.3)
Emotional well-being, median and IQR	0.53 (0-0.95)
Emotional well-being gravity, *n* (%)	
None-to-mild (0.00–0.49)	25 (41.7)
Moderate (0.50–1.00)	27 (45)
Severe-to-very severe (1.01–3.00)	8 (13.3
Social functioning, median and IQR	0.45 (0–1.3)
Social functioning, *n* (%)	
none-to-mild (0.00–0.49)	30 (50)
Moderate (0.50–1.00)	13 (21.7)
Severe-to-very severe (1.01–3.00)	17 (28.3)

UCLA: University of California, Los Angeles Scleroderma Clinical Trial Consortium GIT 2.0; SSc: Systemic Sclerosis; IQR: Interquartile Range. The severity for each reported score was define as “none-to-mild”, “moderate” and “severe-to-very severe”, according to Khanna et al. [31].

#### Comparative analysis of circulating levels of markers of MT, ID and inflammation between SSc patients and HC

**Fig. 1:**
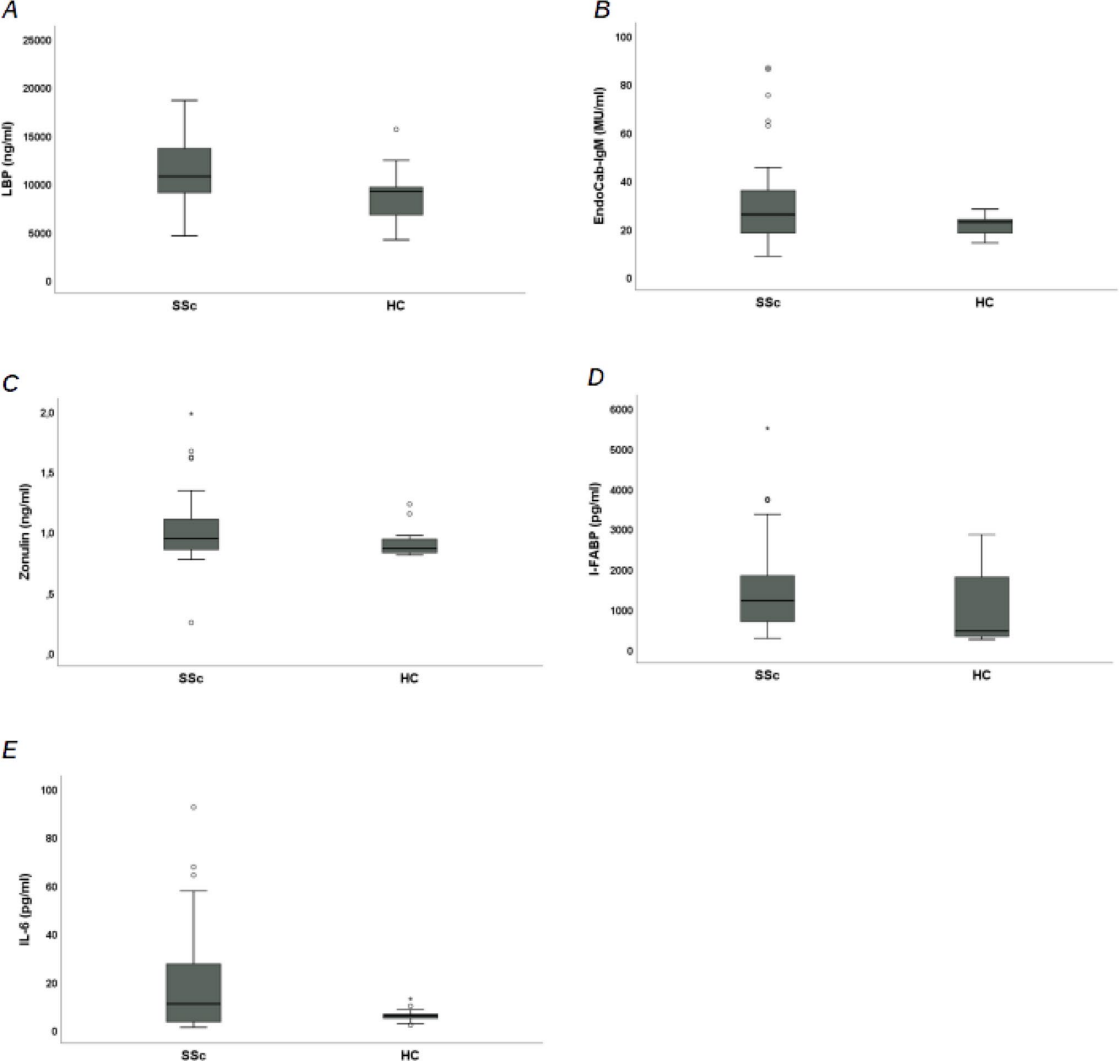
Comparative analysis of circulating levels of markers of microbial translocation (MT), marker of inflammation and markers of intestinal damage (ID) between systemic sclerosis (SSc) patients and healthy controls (HC). **A** Lipopolysaccharide-Binding Protein (LBP); **B** Antiendotoxin Core Antibody (EndoCab) IgM; **C** Zonulin; **D** Intestinal Fatty Acid Binding Protein (I-FABP); **E** Interleukin (IL)-6

SSc patients had a statistically significant higher median value of circulating levels of markers of MT, inflammation and ID than HC (Table 3). Median LBP serum levels were significantly higher in SSc patients compared to HC [10745 ng/ml (IQR 9060–13650) vs. 9209 ng/ml (IQR 6755–9624), *p* < 0.001] (Fig. 1A). Median EndoCab IgM serum levels were significantly higher in SSc patients compared to HC [25.86 MU/ml (IQR 18.18–35.82) vs. 22.70 MU/ml (IQR 18.21–23.80), *p* < 0.05] (Fig. 1B). Median Zonulin serum levels were significantly higher in SSc patients compared to HC [0.94 ng/ml (IQR 0.86–1.11) vs. 0.86 ng/ml (IQR 0.83–0.94), *p* < 0.05] (Fig. 1C). Median I-FABP serum levels were significantly higher in SSc patients compared to HC [1221.50 pg/ml (IQR 697.60–1836) vs. 460.45 pg/ml (IQR 327.75–1802.10), *p* < 0.05] (Fig. 1D). Median IL-6 serum levels were significantly higher in SSc patients compared to HC [10.81 pg/ml (IQR 3.35–27.36) vs. 5.92 pg/ml (IQR 4.80–6.58), *p* < 0.05] (Fig. 1E).

**Table 3:** Comparative analysis of circulating levels of markers of microbial translocation (MT), marker of inflammation and markers of intestinal damage (ID) between systemic sclerosis (SSc) patients and healthy controls (HC)

	SSc (*n* = 60)	HC (*n* = 20)	*p*
LBP, ng/ml, median and IQR	10745 (9060–13650)	9209 (6755–9624)	< 0.001
EndoCab IgM, MU/ml, median and IQR	25.86 (18.18–35.82)	22.70 (18.21–23.80)	< 0.05
IL-6, pg/ml, median and IQR	10.81 (3.35–27.36)	5.92 (4.80–6.58)	< 0.05
I-FABP, pg/ml, median and IQR	1221.50 (697.60–1836)	460.45 (327.75–1802.10)	< 0.05
Zonulin, ng/ml, median and IQR	0.94 (0.86–1.11)	0.86 (0.83–0.94)	< 0.05

SSc: Systemic Sclerosis; HC: Healthy Controls; LBP: Lipopolysaccharide-Binding Protein; EndoCab: Antiendotoxin Core Antibody; IL-6: Interleukin-6; I-FABP: Intestinal Fatty Acid Binding Protein; IQR: Interquartile Range

#### Relationship between circulating levels of markers of MT, ID and inflammation and *UCLA* scores

**Fig. 2:**
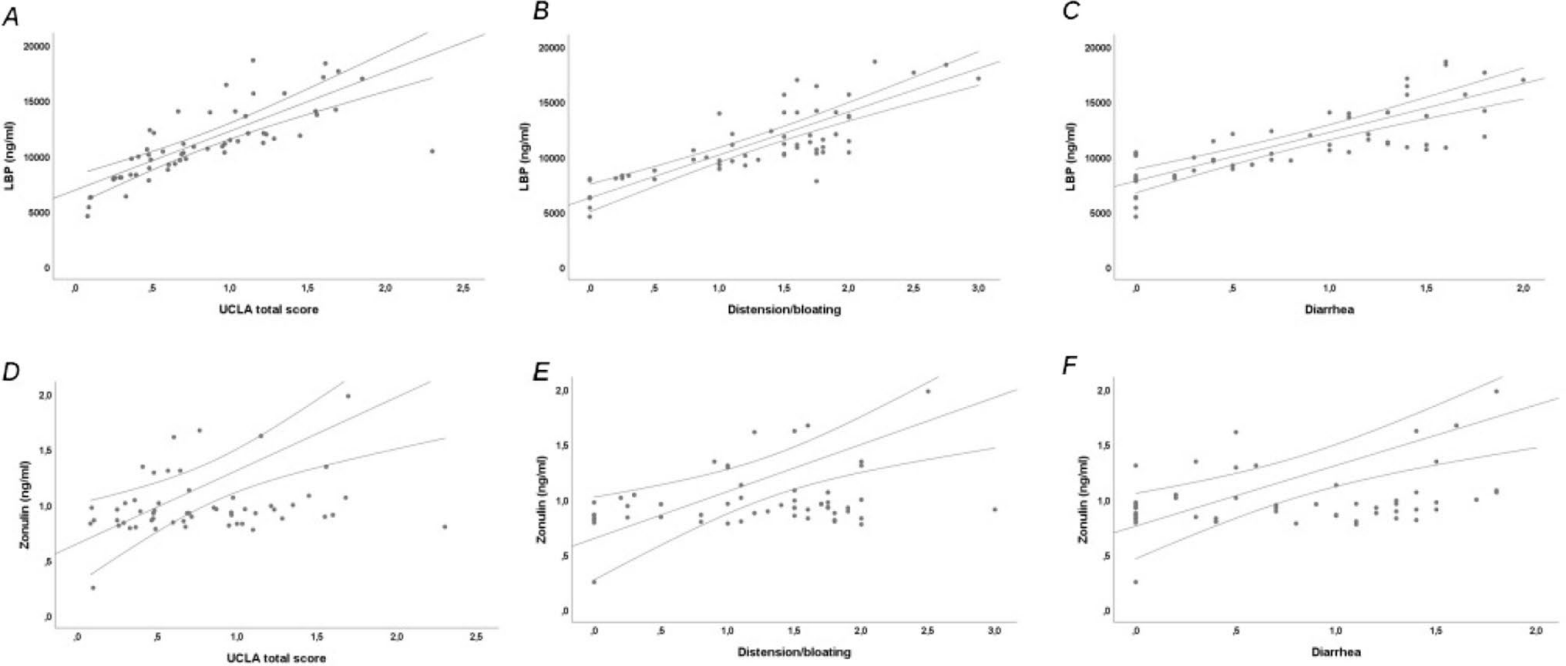
Bivariate correlations between Lipopolysaccharide-Binding Protein (LBP) and Zonulin serum levels and University of California, Los Angeles Scleroderma Clinical Trial Consortium GIT 2.0 (UCLA SCTC GIT 2.0, UCLA) total score, UCLA distension/bloating and UCLA diarrhea in systemic sclerosis (SSc) patients. **A** Positive linear correlation between LBP and UCLA total score (*r* = 0.851, *p* < 0.001); **B** Positive linear correlation between LBP and UCLA distension/bloating (*r* = 0.793, *p* < 0.001); **C** Positive linear correlation between LBP and UCLA diarrhea (*r* = 0.836, *p* < 0.001); **D** Positive linear correlation between Zonulin and UCLA total score (*r* = 0.369, *p* < 0.01); **E** Positive linear correlation between Zonulin and UCLA distension/bloating (*r* = 0.331, *p* < 0.01); **F** Positive linear correlation between Zonulin and UCLA diarrhea (*r* = 0.443, *p* < 0.001)

**Fig. 3:**
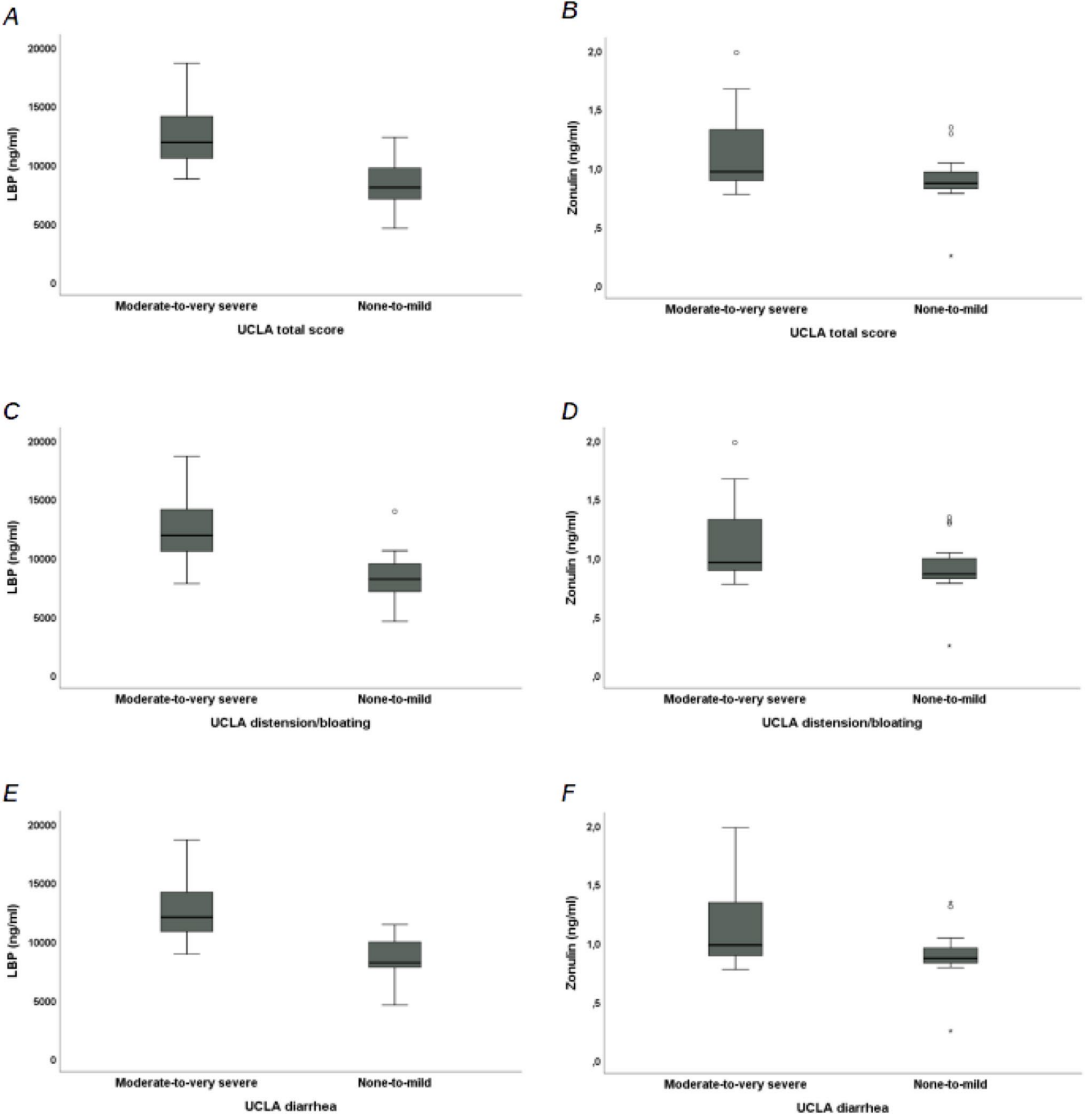
Comparative analysis of Lipopolysaccharide-Binding Protein (LBP) and Zonulin serum level between patients with University of California, Los Angeles Scleroderma Clinical Trial Consortium GIT 2.0 (UCLA SCTC GIT 2.0, UCLA) total score, UCLA distension/bloating and UCLA diarrhea “moderate-to-very severe” or “none-to-mild” 2. **A** LBP between patients with UCLA total score “moderate-to-very severe” or “none-to-mild”; **B** LBP between patients with UCLA distension/bloating “moderate-to-very severe” or “none-to-mild”; **C** LBP between patients with UCLA diarrhea “moderate-to-very severe” or “none-to-mild”; **D** Zonulin between patients with UCLA total score “moderate-to-very severe” or “none-to-mild”; **E** Zonulin between patients with UCLA distension/bloating“moderate-to-very severe” or “none-to-mild”; **F** Zonulin between patients with UCLA diarrhea “moderate-to-very severe” or “none-to-mild”

We found a statistically significant positive linear correlation between LBP and UCLA total score (*r* = 0.851, *p* < 0.001) (Fig. 2A), UCLA distension/bloating (*r* = 0.793, *p* < 0.001) (Fig. 2B), UCLA diarrhea (*r* = 0.836, *p* < 0.001) (Fig. 2C), UCLA fecal soilage (*r* = 0.580, *p* < 0.001) and UCLA constipation (*r* = 0.333, *p* < 0.01). Moreover, a statistically significant positive linear correlation exists between Zonulin and UCLA total score (*r* = 0.369, *p* < 0.01) (Fig. 2D), UCLA distension/bloating (*r* = 0.331, *p* < 0.01) (Fig. 2E) and UCLA diarrhea (*r* = 0.443, *p* < 0.001) (Fig. 2F). We did not find a statistically significant correlation between UCLA total score and I-FABP (*r* = −0.056, *p* > 0.05), EndoCab IgM (*r* = 0.122, *p* > 0.05) or IL-6 (*r* = −0.009, *p* > 0.05). SSc patients who reported an UCLA total score “moderate-to-very severe” had statistically significant higher median LBP [11890 ng/ml (IQR 10540–14105) vs. 8050 ng/ml (IQR 7066–9704), *p* < 0.001] and Zonulin [0.97 ng/ml (IQR 0.89–1.33) vs. 0.87 ng/ml (IQR 0.82–0.96), *p* < 0.05] compared to SSc patients with UCLA total score “none-to-mild” (Fig. 3A-B). SSc patients with UCLA distension/bloating “moderate-to-very severe” had statistically significant higher median LBP [11890 ng/ml (IQR 10540–14105) vs. 8170 ng/ml (IQR 7110–9476), *p* < 0.001] and Zonulin [0.96 ng/ml (IQR 0.89–1.33) vs. 0.86 ng/ml (IQR 0.82–0.99), *p* < 0.05] compared to SSc patients who reported “none-to-mild” symptoms at UCLA distension/bloating (Fig. 3C-D). SSc patients who reported “moderate-to-very severe” symptoms at UCLA diarrhea had statistically significant higher median LBP [12045 ng/ml (IQR 10830–14170) vs. 8170 ng/ml (IQR 7787–9935), *p* < 0.001] and Zonulin [0.98 ng/ml (IQR 0.89–1.34) vs. 0.87 ng/ml (IQR 0.83–0.96), *p* < 0.01] compared to SSc patients with UCLA diarrhea “none-to-mild” (Fig. 3E-F).

#### Relationship between UCLA scores and clinical characteristics of SSc patients

We found a statistically significant positive linear correlation between UCLA total score and DAI (*r* = 0.471, *p* < 0.001) and DSS (*r* = 0.310, *p* < 0.05); between UCLA reflux and DSS (*r* = 0.339, *p* < 0.01); between UCLA distension/bloating and DAI (*r* = 0.357, *p* < 0.01); between UCLA diarrhea and DAI (*r* = 0.399, *p* < 0.01) and DSS (*r* = 0.256, *p* < 0.05); between UCLA fecal soilage and DAI (*r* = 0.433, *p* < 0.001) and DSS (*r* = 0.317, *p* < 0.05); between UCLA constipation and disease duration (*r* = 0.318, *p* < 0.05). We did not find a statistically significant correlation between age and UCLA total score (*r* = −0.003, *p* > 0.05), UCLA reflux (*r* = −0.118, *p* > 0.05), UCLA distension/bloating (*r* = −0.001, *p* > 0.05), UCLA diarrhea (*r* = 0.078, *p* > 0.05), UCLA fecal soilage (*r* = −0.113, *p* > 0.05) or UCLA constipation (*r* = 0.076, *p* > 0.05). Moreover, we did not find a statistically significant correlation between mRSS and UCLA total score (*r* = 0.175, *p* > 0.05), UCLA reflux (*r* = 0.131, *p* > 0.05), UCLA distension/bloating (*r* = 0.175, *p* > 0.05), UCLA diarrhea (*r* = 0.157, *p* > 0.05), UCLA fecal soilage (*r* = 0.153, *p* > 0.05) or UCLA constipation (*r* = 0.076, *p* > 0.05). SSc patients who reported an UCLA total score “moderate-to-very severe” had statistically significant higher median DAI than SSc patients with UCLA total score “none-to-mild” [2.5 (IQR 1.63–4.26) vs. 1.26 (IQR 0.59–2.67), *p* < 0.05]. SSc patients with UCLA distension/bloating “moderate-to-very severe” had statistically significant higher median disease duration compared to SSc patients who reported “none-to-mild” symptoms at UCLA distension/bloating [14 years (IQR 8–24) vs. 9 years (IQR 5–15), *p* < 0.05]. SSc patients who reported “moderate-to-very severe” symptoms at UCLA diarrhea had statistically significant higher median DAI than SSc patients with UCLA diarrhea “none-to-mild” [2.5 (IQR 1.67–4.09) vs. 1.34 (IQR 0.59–3.75), *p* < 0.05].

#### Multivariable models

The logistic regression analysis showed LBP as the only variable associated with an UCLA total score “moderate-to-very severe” [OR 1.001 (CI 95%: 1.001–1.002), *p* < 0.001] (Table 4). The logistic regression analysis showed LBP [OR 1.002 (CI 95%: 1.001–1.003), *p* < 0.01] and disease duration [OR 1.242 (CI 95%: 1.023–1.506), *p* < 0.05] as variables associated with UCLA distension/bloating “moderate-to-very severe” in SSc patients (Table 4). The logistic regression analysis showed LBP as the only variable associated with UCLA diarrhea “moderate-to-very severe” [OR 1.002 (CI 95%: 1.001–1.003), *p* < 0.01] (Table 4).

**Table 4:** Stepwise logistic regression analysis showing the association between University of California, Los Angeles Scleroderma Clinical Trial Consortium GIT 2.0 (UCLA SCTC GIT 2.0, UCLA) total score “moderate-to-very severe” or UCLA distension/bloating “moderate-to-very severe” or UCLA diarrhea “moderate-to-very severe” and independent variables

	OR (CI 95%)	*P*
UCLA total score “moderate-to-very severe”		
LBP, ng/ml	1.001 (1.001–1.002)	< 0.001
Zonulin, pg/ml	4.860 (0.097–243.802)	> 0.05
DAI	0.858 (0.349–2.112)	> 0.05
DSS	1.215 (0.634–2.327)	> 0.05
UCLA distension/bloating score “moderate-to-very severe”		
LBP, ng/ml	1.002 (1.001–1.003)	< 0.01
Zonulin, pg/ml	5.277 (0.077–363.966)	> 0.05
Disease duration, years	1.242 (1.023–1.506)	< 0.05
DAI	0.894 (0.475–1.682)	> 0.05
UCLA diarrhea “moderate-to-very severe”		
LBP, ng/ml	1.002 (1.001–1.003)	< 0.01
Zonulin, pg/ml	13.173 (0.098–1771.742)	> 0.05
DAI	0.550 (0.179–1.688)	> 0.05
DSS	1.694 (0.706–4.063)	> 0.05
Disease duration, years	1.092 (0.950–1.256)	> 0.05

LBP: Lipopolysaccharide-Binding Protein; DAI: Disease Activity Index; DSS: Disease Severity Scale; OR: Odds Ratio; CI: Confidence Interval

## Discussion

In this cross-sectional study SSc patients showed significantly higher levels of circulating markers of MT, inflammation and ID than HC. Changes in intestinal permeability promote dysbiosis which in turn alter the intestinal barrier leading to immunological activation and MT [25].

MT, defined as the passage of indigenous bacteria or their products from the gastrointestinal tract through the lamina propria to the mesenteric lymph nodes and other organs, occurs in relation to changes in mucosal integrity induced by disruption of the intestinal epithelial barrier function, intestinal bacterial overgrowth and changes in the composition of bacterial microbes in the gut, all conditions present during SSc.

So far, ID and MT have been demonstrated to occur in several infectious and noninfectious conditions [7–20]. They indeed may represent a possible trigger for immune system activation, inflammation and thrombosis in SARS-CoV2 infection [9, 13, and 14], HIV infection [15], community-acquired pneumonia [16], liver [17] and cardiovascular diseases [18–20], may be considered as early biomarkers of subsequent bloodstream infection in *Clostridioides difficile* infection (CDI) [7, 8] or infection’s severity in SARS-CoV2 infection [9, 13] or as indirect biomarkers of gut barrier dysfunction possibly influencing the risk of infection following endoscopic surgery [32]. Methods for determining gut barrier integrity, such as histological findings of intestinal biopsies or oligosaccharide absorption assays, are difficult to assess in clinical practice. For this reason, the evaluation of novel serum biomarkers is useful and effective in disease evaluation and play an essential role in monitoring the clinical complications of GI tract. Several biomarkers in clinical practice have been evaluated for assessing MT and ID, among which LPB, EndoCab IgM, I-FABP and Zonulin are the most studied. LBP is an acute-phase reaction protein produced by hepatocytes and binds to bacterial LPS deriving mostly from translocation from the intestine and thus representing both a marker of endotoxemia and increased intestinal permeability [10, 25]; at the same time, the presence of circulating LPS induces the consumption of EndoCab. I-FABP is a protein exclusively presents in the cytoplasm of enterocytes in the small intestine and usually its passage into circulation is minimal, while its levels increased in the blood when damage to the intestinal epithelium occurs. Zonulin is an acute-phase reaction protein that controls intestinal permeability by decreasing the stability of tight junctions. Dysregulation of Zonulin levels can lead to increased intestinal permeability, allowing for the translocation of bacteria and toxins into the bloodstream, triggering an immune response and promoting inflammation [10]. In fact, MT can exacerbate systemic inflammation leading to an increase of IL-6.

With regard to SSc, to the best of our knowledge, only one study analyzed the degree of intestinal permeability; Authors found that SSc patients had a significantly increased concentration of LPS than non-SSc subjects, while no difference was found for I-FABP. Conversely, in our report we could demonstrate that patients with SSc exhibit not only a higher level of MT, but also ID and systemic inflammation than controls, suggesting that increased intestinal permeability and gut barrier alteration have a role in the pathogenesis of the disease.

In SSc bacterial overgrowth determines malabsorption of fat and vitamins with concomitant production of gas and osmotically active products, which in turn lead to abdominal tenderness, diarrhea and bloating. SIBO is due to delayed gastric emptying and prolonged orofaecal transit time and is characterized by an increase in the number and/or an abnormal type of bacteria, which may count up to 60% higher than in HC [33].

In SSc, the most frequent changes in gut microbial composition are characterized by reduced number of beneficial commensal genera (e.g., *Faecalibacterium*, *Clostridium*, *Rikenella* and *Bacteroides*) and increased number of pathobiont genera (e.g., *Fusobacterium* spp*, Prevotella* spp*, Lactobacillus* spp*, Ruminococcus* spp*, Akkermansia* spp*, Erwinia* spp *and Trabsulsiella* spp) [34]. Several studies found that, in SSc patients with severe GI tract involvement, some genera, such as *Fusobacterium* spp, are more represented in the colonic lavage specimen [35]. Recently, has been demonstrated that microbiomes of IgG4-related diseases and SSc patients distinctly separated from those of HC, with abundance of opportunistic pathogenic *Clostridium* and typically oral *Streptococcus* spp [36]. Moreover, the most differentially abundant taxa were not the facultative anaerobes consistently identified in inflammatory bowel diseases, suggesting the microbial signatures of these cohorts of patients do not result from mucosal inflammation [36]. The results of our study, according to these findings, suggest that GI involvement in SSc patients is not inflammatory but is due to alterations of the mucosa and intestinal transit with consequently MT.

Of interest, SSc patients with moderate-to-severe GI tract symptoms, in particular bloating/distention and diarrhea, showed higher *Prevotella* spp levels compared with patients with none-to-mild GI tract symptoms [34]. It may therefore be hypothesized that the alteration of SSc patients’ microbiota may be associated with a gut mucosal impairment, as it has been described during CDI [7].

Severe gastrointestinal symptoms are associated with poor QoL, more severe disease leading to impaired QoL and malnutrition [37, 38]. Since, symptoms of GI dysfunction are difficult to evaluate in SSc, UCLA questionnaire is the most validated tool to characterized the GI tract involvement [17].

We found a statistically significant positive correlation between items of UCLA including distension/bloating, diarrhea, fecal soilage, constipation and LBP.

Patients with higher UCLA total score showed more activity and severity of disease. Moreover, patients with UCLA total score “moderate-to-very severe” showed higher levels of LBP when compared to patients with UCLA total score “none-to-mild”. In this score that represents a more severe GI tract involvement, it is interesting to note how diarrhea and distention/bloating are the items significatively related to MT and ID. Since, SSc is characterized by severe and untreatable GI involvement with changes of the fecal microbiota composition is reasonable to observe high levels of LBP associated with GI complications.

Patients with higher UCLA total score showed more activity and severity of disease. Moreover, patients with UCLA total score “moderate-to-very severe” showed higher levels of LBP when compared to patients with UCLA total score “none-to-mild”. In this score that represents a more severe GI tract involvement, it is interesting to note how diarrhea and distention/bloating are the items significatively related to MT and ID.

Bacteria producing gas and osmotically active by-products can promote diarrhea and are able to damage intestinal mucosa modifying intestinal microbiota promoting inflammation and dysbiosis [39].

At logistic regression analysis LBP was the only biomarker associated with UCLA total score “moderate-to-very severe”. Also, LBP was associated with disease duration and UCLA distension/bloating “moderate-to-very severe” and, finally, LBP was the only variable associated with UCLA diarrhea “moderate-to-very severe”.

As for clinical implications, the results of the present study showed how markers of MT, especially LBP, are associated with alteration of gut permeability in SSc patients. With this background, markers of ID and MT might represent useful tools for the monitoring of mucosal integrity in patients with SSc and to identify patients with higher severity of gut disturbance. These findings may have important clinical consequences, since new therapeutic strategies aiming at reducing LPB levels (e.g., probiotics) may ameliorate patients’ GI symptoms. Unfortunately, we could not find similar association when analyzing the other biomarkers, suggesting that LBP more than the other biomarkers may have important clinical implication in the disease. Nevertheless, the little sample size may also have contributed to the possible underestimation of a relation between the other biomarkers and clinical aspects of the disease, suggesting the need of additional studies.

The present study has several limitations, such as (i) it is a single-center study; (ii) the sample size is small, since SSc is a rare disease; and (iii) fecal microbiota was not assessed. Despite this, we firmly believe that this study paves the way for new researches towards a better understanding of the pathogenesis of SSc.

Overall, the dysregulation gut mucosal integrity expressed by high levels of MT and ID biomarkers in patients with SSc highlights the complex interplay between the immune system, gut barrier function, and fibrotic processes in the pathogenesis of this debilitating disease. Further studies may provide valuable insights into the underlying mechanisms of the GI complications related to SSc and help to identify new treatment strategies.

## Data Availability

No datasets were generated or analyzed during the current study.
